# Effect of astragaloside IV on indoxyl sulfate-induced kidney injury in mice via attenuation of oxidative stress

**DOI:** 10.1186/s40360-018-0241-2

**Published:** 2018-09-03

**Authors:** Chunlan Ji, Yueming Luo, Chuan Zou, Lihua Huang, Ruimin Tian, Zhaoyu Lu

**Affiliations:** National Key Unit of Clinical Research of TCM on Chronic Kidney Diseases, Key Unit of Kidney Diseases, Guangdong Provincial Hospital of Chinese Medicine, The Second Clinical College of Guangzhou University of Chinese Medicine, 111th Dade Road, Guangzhou, 510120 People’s Republic of China

**Keywords:** Astragaloside IV, IS, Tubulointerstitial injury, Oxidative stress

## Abstract

**Background:**

*Astragalus membranaceus*, a traditional Chinese medicine (TCM), has been widely used in the treatment of chronic kidney disease (CKD) in China. Astragaloside IV is one of the major compounds of *Astragalus membranaceus*. Recent research has shown that astragaloside IV demonstrates pharmacological effects, such as anti-inflammatory, anti-fibrotic and anti-oxidative stress activities. Our aim was to investigate the effects of astragaloside IV on indoxyl sulfate (IS)-induced kidney injury in vivo, and to study the underlying mechanism.

**Methods:**

Forty C57BL/6 mice with ½ nephrectomy were divided into four groups: control group (*n* = 10), IS group (*n* = 10), IS plus 10 mg/kg of astragaloside IV group (*n* = 10) and IS plus 20 mg/kg of astragaloside IV group (*n* = 10). IS intraperitoneal injection and astragaloside IV treatment were administered continuously for 1 month. Next, the blood urea nitrogen (BUN) level, serum IS level, tubulointerstitial injury, renal oxidative stress and inflammatory injury were assessed.

**Results:**

The IS intraperitoneal injection mouse group showed increasing levels of serum IS, BUN, tubulointerstitial injury, renal oxidative stress and inflammatory injury. Astragaloside IV treatment couldn’t reduce the serum IS level or renal nuclear factor-κB and interleukin-1β levels. However, 20 mg/kg astragaloside IV treatment reduced the BUN level and significantly attenuated IS-induced tubulointerstitial injury. Renal oxidative stress was decreased by the administration of astragaloside IV.

**Conclusions:**

These results suggest that astragaloside IV prevents IS-induced tubulointerstitial injury by ameliorating oxidative stress and may be a promising agent for the treatment of uremia toxin-induced injury.

**Electronic supplementary material:**

The online version of this article (10.1186/s40360-018-0241-2) contains supplementary material, which is available to authorized users.

## Background

In chronic kidney disease (CKD), metabolic changes, disorders of gut microflora and impaired urinary excretion of metabolites lead to the accumulation of uremic toxins in the body [1]. Indoxyl sulfate (IS), one of the most extensively studied uremic toxins, has been increasingly recognized as an important contributor to kidney and heart dysfunction [2–4].

The pathophysiologic importance of protein-bound uremic toxins has been neglected for a long time [5, 6]. Dialysis is not powerful enough to remove protein-bound uremic toxins alone [7, 8]. Removing IS is very difficult in CKD patients. Consequently, identifying effective measures to reduce IS-induced injury is of significant value.

Indeed, the mechanism by which IS induces its toxic effects has now been clarified. Previous studies have demonstrated IS-induced tubulointerstitial injury in patients with CKD via the induction of oxidative stress and activation of the nuclear factor (NF)-κB pathway, producing various cytokines and inflammatory mediators, thereby promoting kidney damage [9–11].

*Astragalus membranaceus*, a traditional Chinese medicine (TCM), has been widely used for the treatment of CKD in China [12]. Astragaloside IV is one of the major compounds of *Astragalus membranaceus*. Astragaloside IV exerts considerable pharmacological effects, such as anti-inflammatory, anti-fibrotic and anti-oxidative stress activities [13, 14]. Although the wide use of *Astragalus* in CKD, and the antioxidant and anti-inflammatory properties of astragaloside IV, have been well demonstrated, studies regarding its efficacy in the prevention and attenuation of IS-induced tubulointerstitial injury have not been undertaken. Based on previous research, we hypothesize that astragaloside IV may affect IS-induced tubulointerstitial injury. Therefore, the aim of this study was to evaluate the effect of astragaloside IV on IS-induced tubulointerstitial injury in vivo, and to study its mechanism of action.

## Methods

### Animals, reagents and experimental drugs

Forty eight-week-old male C57BL/6 mice were used for this study. The C57BL/6 mice were purchased from Beijing Vital River Laboratory Animal Technology Company (Beijing, China). Each mouse was housed in our animal facility under pathogen-free conditions and was fed a standard laboratory diet, with free access to water.

Astragaloside IV (purity > 99%) was provided by Shanghai Winherb Medical Technology (Shanghai, China). IS potassium salt (purity 99.8%) and anti-β-actin antibody were obtained from Sigma (St. Louis, MO, USA). Anti-8-OHdG and anti-NF-κB were procured from Abcam (Cambridge, UK). Anti-cytochrome C was purchased from Santa Cruz Biotechnology (Santa Cruz, CA, USA). Fluorescein isothiocyanate (FITC)-conjugated anti-mouse immunoglobulin G (IgG), cy3-conjugated anti-rabbit IgG, horseradish peroxidase (HRP)-conjugated anti-mouse and rabbit IgG were purchased from Beyotime Biotechnology Company (Jiangsu, China).

### Establishment of an animal model

The IS-induced tubulointerstitial injury model was carried out as described previously [15]. Forty C57BL/6 mice all underwent nephrectomy. All mice were anesthetized with an intraperitoneal injection of 2.0% pentobarbital sodium (30 mg/kg body weight). Next, the mice were randomly divided into four groups: control group (*n* = 10), IS group (*n* = 10), IS plus 10 mg/kg astragaloside IV group (*n* = 10), and IS plus 20 mg/kg astragaloside IV group (*n* = 10).

The experimental mice received an intraperitoneal injection of IS at a dosage of 100 mg/kg/day for 1 month. The control mice received daily phosphate-buffered saline (PBS) injection at the same volume. Astragaloside IV was suspended in 1% carboxymethyl cellulose (CMC) solution. Astragaloside IV group mice received astragaloside IV treatment by oral simultaneously at a dosage of 10 mg/kg or 20 mg/kg daily for 1 month. IS group mice received 1% CMC solution by oral at the same volume.

At the end of the study, all mice were anesthetized with an intraperitoneal injection of 2.0% pentobarbital sodium and then sacrificed. The sacrifice was implemented by the method of rapid decapitation and then collected the abdominal aorta blood and kidney sample. Blood were centrifugation at 3000 rpm for 15 min to take serum. The blood urea nitrogen (BUN) level, serum IS level, tubulointerstitial injury, renal oxidative stress and inflammatory injury were assessed.

The kidney was decapsulated and divided into several parts. One part was fixed in 10% formalin/PBS and processed for histopathological analysis, while another part was fixed in optimum cutting temperature (OCT) compound and stored at − 80 °C for immunofluorescence (IF) analysis. The remaining kidney tissue were isolate, quickly frozen in liquid nitrogen and stored at − 80 °C for Western-blot and ELISA detection The serum levels of BUN were measured using an auto-biochemical analyzer.

### Indoxyl sulfate measurement

The serum IS concentration was detected by high-performance liquid chromatography (HPLC) tandem mass spectrometry, as described in our previous study [16].

The detection chemical included the standard IS potassium salt (purity 99.8%) and internal standard of hydrochlorothiazide (purity 99.5%), and was obtained from the Institute for Drug Control (Guangzhou, China).

Briefly, the serum samples were deproteinized by the addition of three parts of methanol to one part of serum for the determination of IS. All analyses were performed using an Acquity HPLC system (Agilent Technologies, Santa Clara, CA, USA) and an API3000 triple quadrupole mass spectrometer (Applied Biosystems, Foster City, CA, USA). Separation was performed using a reversed-phase HPLC ZORBAX Eclipse XDB-C18 column (Narrow-Bore 2.1 × 150 mm, 3.5-μm; Agilent). All data were assessed using Analyst14.1 software (Applied Biosystems). Standard curves were constructed using the linear regression formula y = 0.0665 × − 0.0294, R^2^ = 0.9995. The limit of quantification (LOQ) was defined as the lowest concentration for which acceptable precision and accuracy could be guaranteed (< 20%). The limit of detection (LOD) was 0.25 μg/ml.

### Renal histopathological studies

Kidney slices were fixed in 10% formalin/PBS and embedded in paraffin. Sections of 2-μm thickness were prepared and stained with hematoxylin and eosin to evaluate the renal tubulointerstitial injury level. Renal histopathology studies were carried out under an Olympus BX61 microscope equipped with an Olympus DP72 digital camera.

### Immunofluorescence staining

Sections were fixed in 4% paraformaldehyde for 15 min and washed with PBS twice for 10 min. Next, the sections were blocked in 10% casein/PBS (Vector, Burlingame, CA, USA) in PBS for 30 min. The sections were then incubated with primary antibodies overnight in a moisture chamber and washed sufficiently with PBST to remove unbound antibody. Next, the sections were incubated with secondary antibody, FITC-conjugated anti-mouse IgG or cy3-conjugated anti-rabbit IgG for 60 min at room temperature, and were washed as described for the primary antibody. Finally, the sections were stained with 4′,6-diamidino-2-phenylindole solution, washed for 5 min and then mounted on glass slides and analyzed under an Olympus BX61 fluorescence microscope equipped with an Olympus DP72 digital camera (Tokyo, Japan).

### Western blot analysis

Kidney tissues were lysed in RIPA buffer. The lysates were clarified by centrifugation at 12,000 rpm for 30 min at 4 °C, and the protein concentration in each lysate was determined using the BCA Protein Assay Kit (Pierce, Rockford, IL, USA). The protein samples were separated by sodium dodecyl sulfate–polyacrylamide gel electrophoresis and electroblotted onto nitrocellulose membranes. The membranes were blocked with 10% casein/PBS, incubated with a primary antibody at 4 °C overnight, and then incubated with HRP-conjugated secondary antibody. Immunoreactive bands were visualized using enhanced chemiluminescent reagent and exposure to the Bio-Rad ChemiDoc XRS+ system (Hercules, CA, USA).

### Enzyme-linked immunosorbent assay (ELISA) for interleukin (IL)-1β and IL-6

The concentrations of IL-1β and IL-6 in kidney tissues were determined using mouse IL-1β and IL-6 ELISA kits (R&D Systems, Minneapolis, MN, USA) according to the manufacturer’s instructions.

### Evaluation of 8-hydroxy-2′-deoxyguanosine (8-OHdG) expression and malondialdehyde (MDA) content, as well as superoxide dismutase (SOD) activity, in renal tissue

The expression of 8-OHdG, a marker of oxidative stress [17], in the kidney was examined by IF using anti-8-OHdG. The MDA level was assayed using the Lipid Peroxidation MDA Assay Kit (S0131; Beyotime, Nanjing, China). SOD activity was assayed using the Total Superoxide Dismutase Assay Kit (S0101; Beyotime) following the manufacturer’s instructions.

### Statistics

All data analyses were performed using SPSS software (ver. 13.0; SPSS, Inc., Chicago, IL, USA). The data are expressed as means ± standard error (SE). Comparisons among groups were conducted using analysis of variance. A value of *P* < 0.05 was deemed to indicate statistical significance.

## Results

### Astragaloside IV cannot reduce the serum levels of indoxyl sulfate in IS intraperitoneal injection mice

**Fig. 1:**
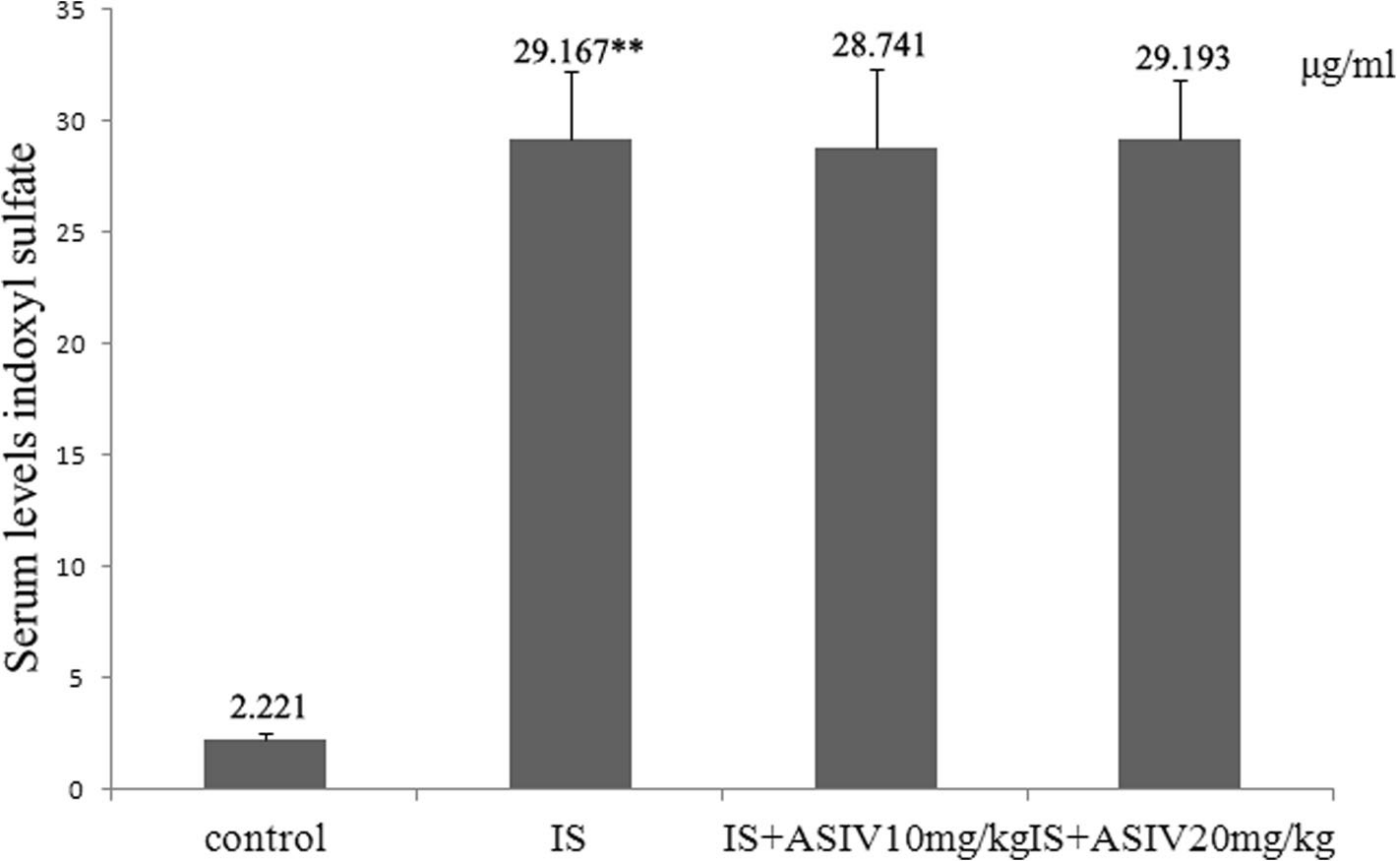
Serum levels of indoxyl sulfate. The serum indoxyl sulfate concentration of mice was detected by high-performance liquid chromatography (HPLC) tandem mass spectrometry. Data were presented as means ± SE. **P < 0.001 vs. control group. IS: indoxyl sulfate. ASIV: astragaloside IV

In our study, the IS intraperitoneal injection group mice showed a significantly increased serum level of IS compared with that in control group mice. The administration of astragaloside IV did not significantly decrease the serum levels of IS (Fig. 1).

### Astragaloside IV inhibits tubulointerstitial injury in indoxyl sulfate intraperitoneal injection mice

**Fig. 2:**
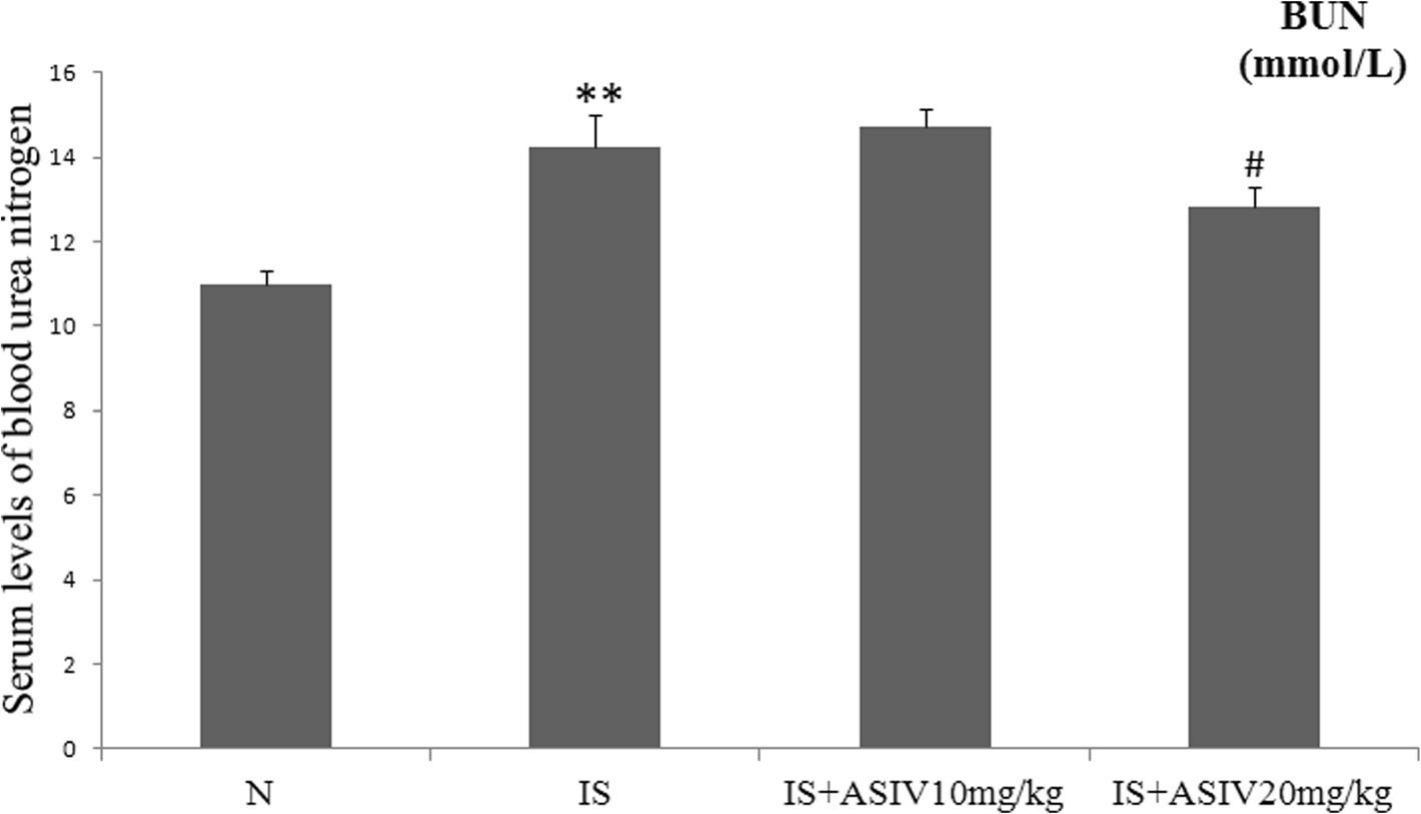
Serum levels of blood urea nitrogen (BUN). IS: indoxyl sulfate. ASIV: astragaloside IV. **P < 0.001 vs. control group. ^#^*P* = 0.048 vs. IS group

**Fig. 3:**
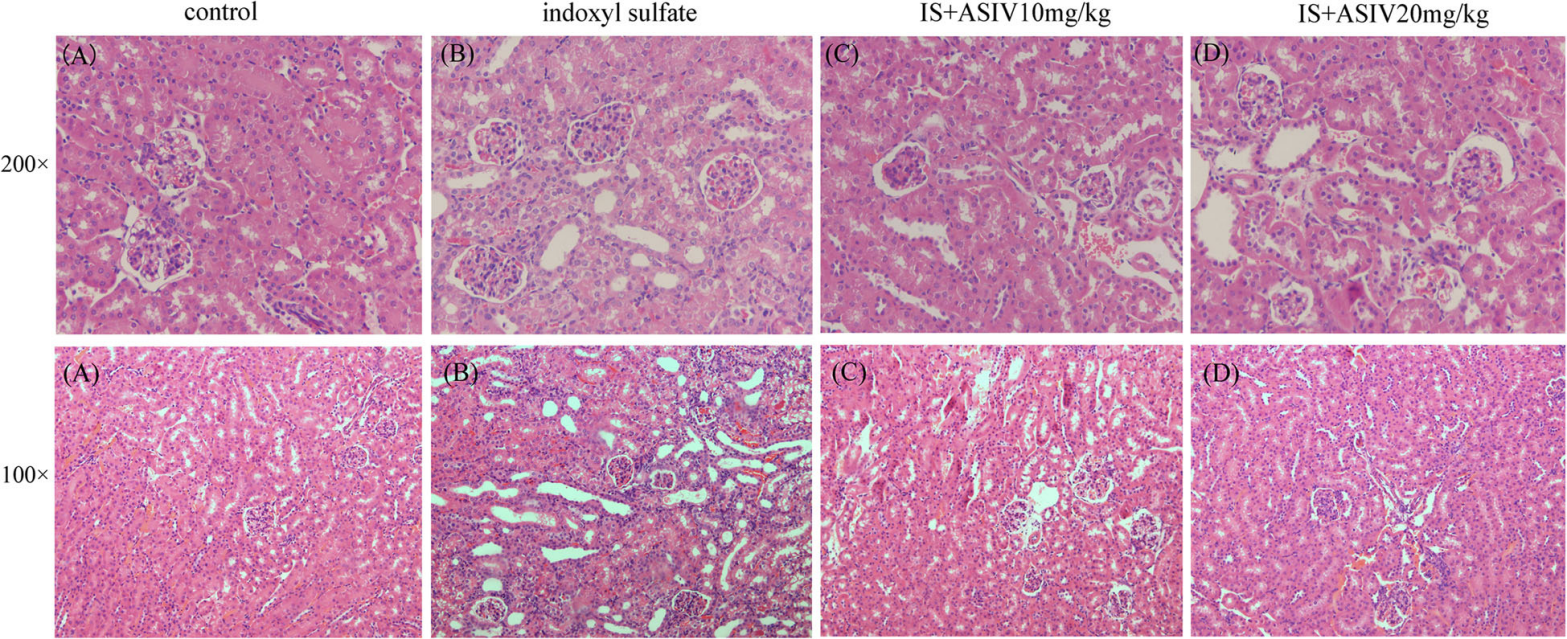
Representative histopathology in IS intraperitoneal injection mice treated with or without astragaloside IV (HE staining). IS intraperitoneal injection mice showed partial tubular epithelial cell degeneration, loss of the brush border, enlargement of the tubular lumen and interstitial expansion. **a**: control group; **b**: IS group; **c**: IS plus 10 mg/kg astragaloside IV treatment group; **d**: IS plus 20 mg/kg astragaloside IV treatment group

The control group mice showed low levels of serum BUN and normal glomerular and tubulointerstitial morphology. The level of serum BUN was significantly increased in IS intraperitoneal injection group mice (*P* < 0.001). In addition, IS intraperitoneal injection mice showed partial tubular epithelial cell degeneration, loss of the brush border, enlargement of the tubular lumen and interstitial expansion (Figs. 2 and 3).

The level of BUN of IS intraperitoneal injection mice was significantly decreased after 20 mg/kg of astragaloside IV treatment (*P* = 0.048). The administration of 20 mg/kg astragaloside IV attenuated the histopathological changes in IS intraperitoneal injection mice (Figs. 2 and 3). Astragaloside IV (10 mg/kg) did not exhibit any effect.

### Astragaloside IV cannot ameliorate renal inflammatory injury in indoxyl sulfate intraperitoneal injection mice

**Fig. 4:**
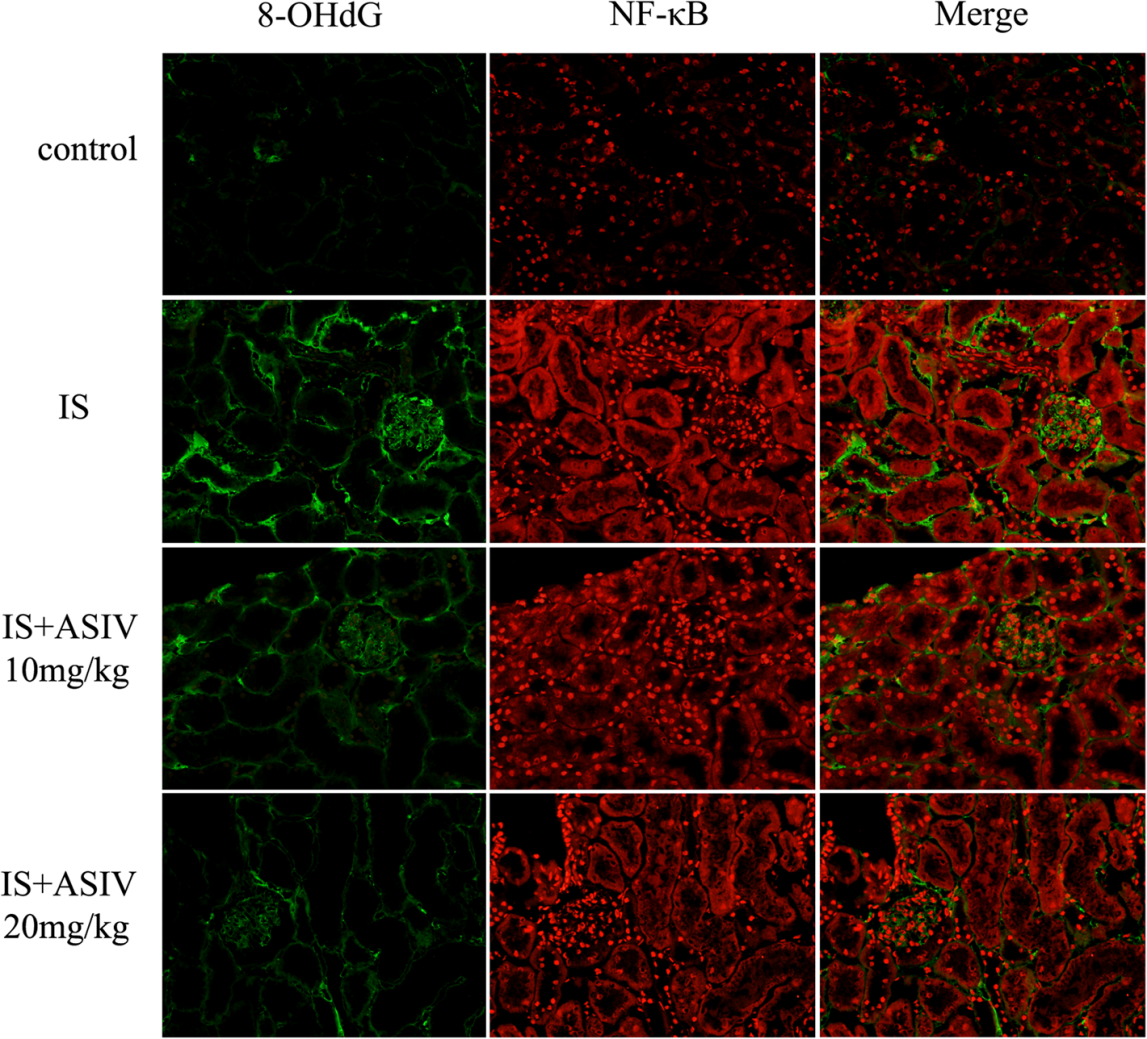
Expression levels of 8-hydroxy-2′-deoxyguanosine (8-OHdG) and nuclear factor (NF)-κB in frozen sections of kidney. Localization of 8-OHdG and NF-κB in frozen sections was determined by immunofluorescence (IF) (× 200) with 8-OHdG antibody (green) and NF-κB ant (red)

**Fig. 5:**
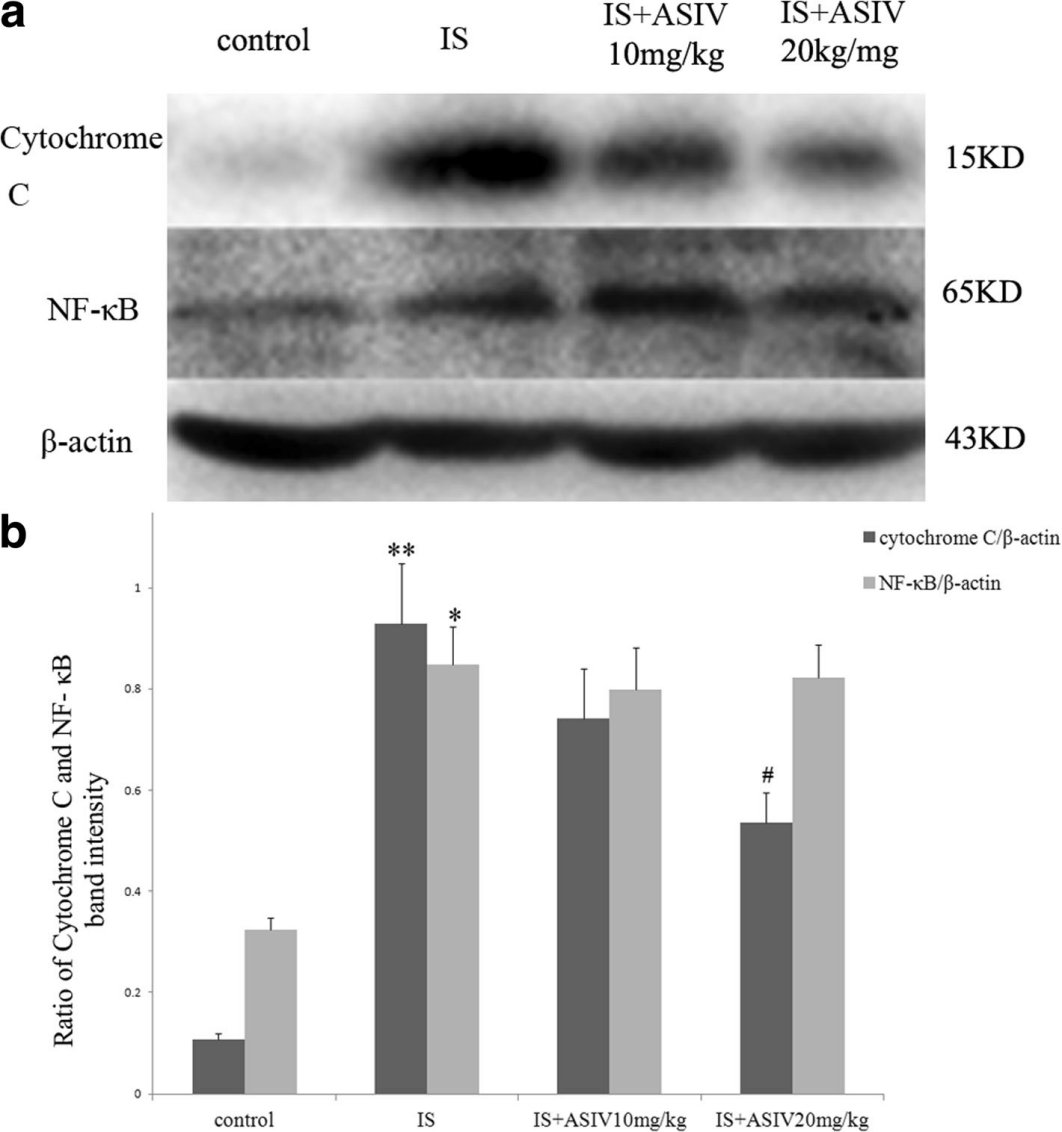
Expression of NF-κB and cytochrome C in kidney tissue. Western blot analysis of the NF-κB and cytochrome C protein levels. **a** Representative western blot of NF-κB and cytochrome C in control and IS mice treated with or without astragaloside IV. **b** The data was shown as ratio of NF-κB or cytochrome C density to β-actin density. The values were expressed as mean ± SE and were compared by analysis of variance. *P = 0.003 vs. control group, **P < 0.001 vs. control group. ^#^*P* = 0.011 vs. IS group

**Fig. 6:**
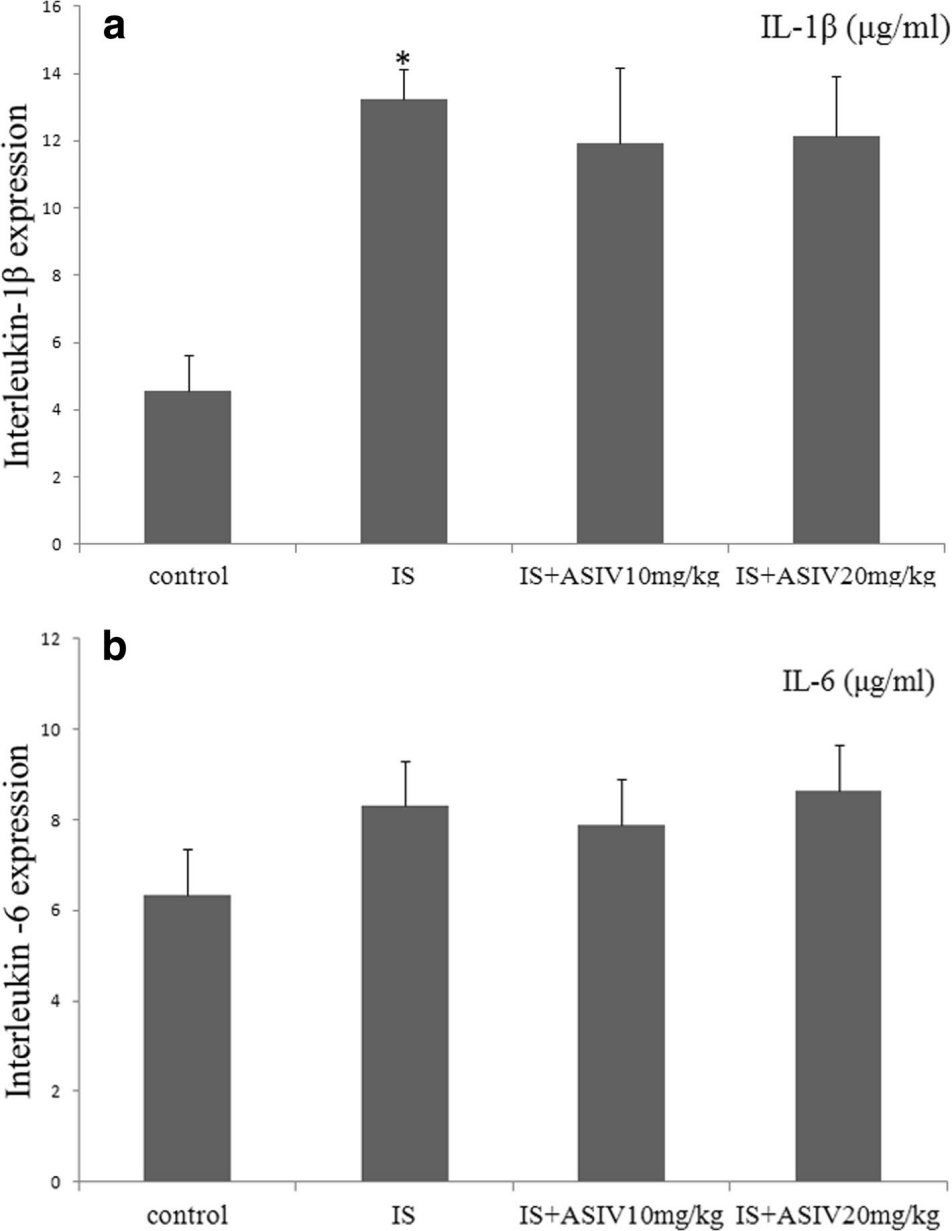
Expression of IL-1β and IL-6 in kidney tissue. Expression of IL-1β and IL-6 protein in the kidney determined with Enzyme-linked immunosorbent assay (ELISA) in IS mice treated with or without astragaloside IV. **a** ELISA analysis of IL-1β in kidney tissues. **b** ELISA analysis of IL-6 in kidney tissues. *P = 0.016 vs. control group

Renal inflammatory injury was evaluated based on the expression of NF-κB, IL-1β, and IL-6. The expression of NF-κB was detected by western blotting and IF staining. The expression level of NF-κB was increased in both glomeruli and tubules in the IS group mice (Figs. 4 and 5). In addition, ELISA showed significantly increased levels of IL-1β expression in kidney homogenates (Fig. 6). The level of IL-6 expression was a bit increased in IS group mice (Fig. 6).

Astragaloside IV treatment could not attenuate the NF-κB expression in glomeruli and tubules. The expression of IL-1β and IL-6 in the astragaloside IV treatment group mice also showed no significant difference compared with that in IS group mice (Figs. 4, 5 and 6).

### Astragaloside IV ameliorates renal oxidative stress in indoxyl sulfate intraperitoneal injection mice

Renal tissue oxidative stress was evaluated by 8-hydroxy-2′-deoxyguanosine (8-OHdG) IF staining, a critical biomarker of oxidative stress, measurement of the MDA level, and the SOD activity assay.

**Fig. 7:**
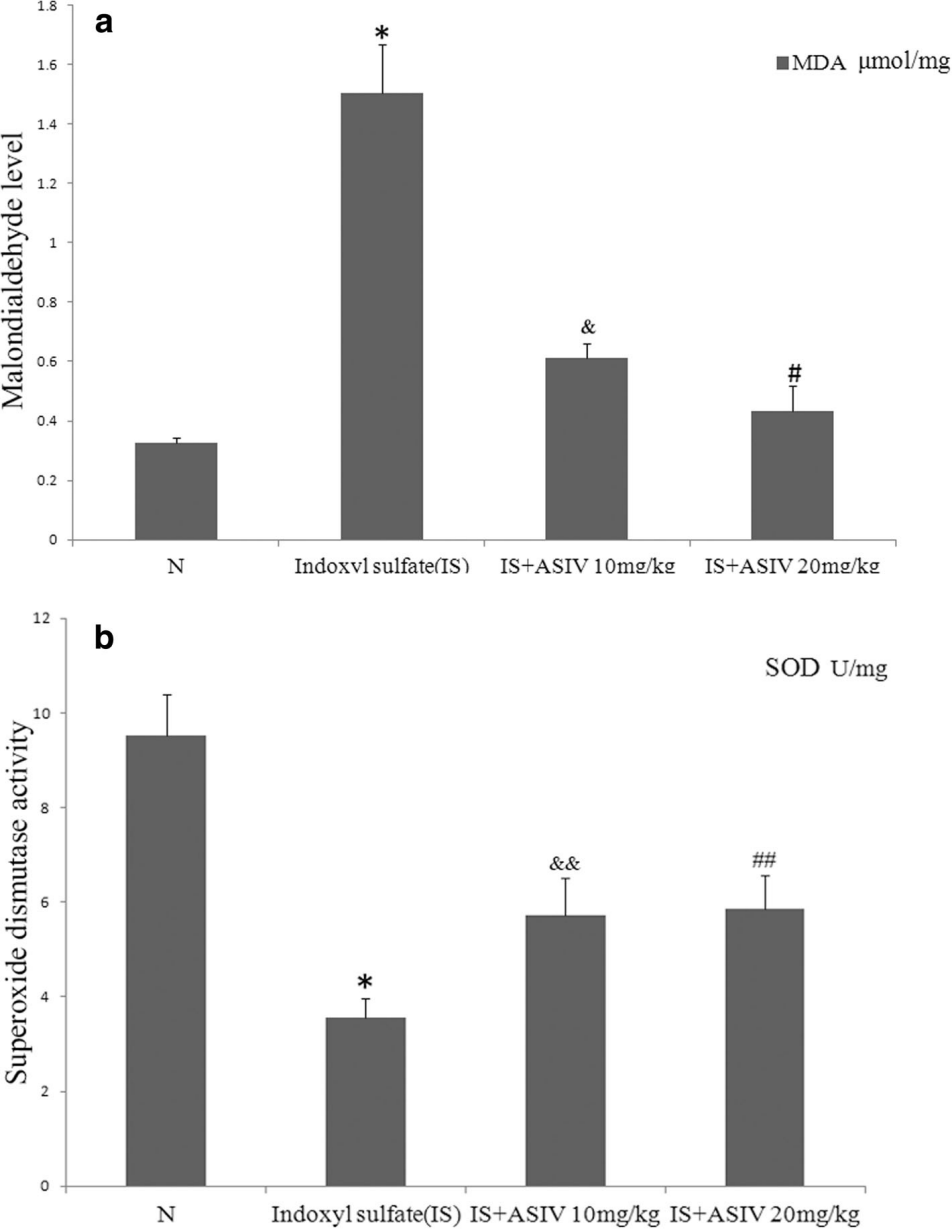
Malondialdehyde (MDA) and superoxide dismutase (SOD) activity assays. Kidney oxidative stress was determined with MDA and SOD activity assays. **a** MDA levels of kidney tissues. **b** SOD activity of kidney tissues. IS group mice showed higher MDA and lower SOD values than control group mice. *P < 0.001 vs. the control group. The MDA value was significantly lower, and SOD activity was significantly higher, after treatment with 20 mg/kg of astragaloside IV. ^#^*P* = 0.036 vs IS group, ^&^*P* = 0.068 vs IS group, ^&&^*P* = 0.043 vs IS group, ^##^*P* = 0.031 vs IS group

IS group mice showed increased 8-OHdG-positive areas in both glomeruli and tubules (Fig. 4). Increased 8-OHdG staining was associated with an increased MDA level and decreased SOD activity in the kidney (Fig. 7).

Astragaloside IV treatment can attenuate 8-OHdG expression in both glomeruli and tubules. In addition, 20 mg/kg of astragaloside IV decreased the MDA level and enhanced SOD activity (Figs. 4 and 7).

Cytochrome C is a marker linking mitochondrial oxidative stress and apoptosis [18]. In our study, the expression of cytochrome C was also increased in IS group mice. In addition, treatment with 20 mg/kg of astragaloside IV significantly attenuated cytochrome C expression (Fig. 5).

## Discussion

In the present study, the protective effect of astragaloside IV against IS-induced tubulointerstitial injury was investigated. Our results showed that astragaloside IV could reduce serum levels of BUN, inhibit renal tubulointerstitial injury and ameliorate renal oxidative stress in IS intraperitoneal injection mice. The evidence supported the effect of astragaloside IV on IS-induced tubulointerstitial injury.

CKD is recognized as a worldwide health problem, and the roles of individual cardiovascular risk factors in CKD are important [19].

Recent studies have found that the colon is an major organ in which uremic toxins are generated. In CKD patients, the imbalance mainly occurs in the colon, presenting as decreased levels of probiotics such as *Lactobacillus* and *Bifidobacteria* and increased pathogenic bacteria such as *Escherichia coli* and *Enterococcus* [20, 21]. Under these conditions, bacteria (mostly *E. coli*) produce numerous toxins from glycolysis of the retained proteins. For example, a fraction of tryptophan is metabolized into indole by *E. coli* in the colon, which is absorbed into blood through the intestinal wall and metabolized into IS in the liver [22].

Uremic toxins such as IS are protein-bound toxins with a molecular weight greater than 200 Da that are difficult to remove [23]. Dialysis is not powerful enough to remove protein-bound uremic toxins alone [7, 8]. Removing IS is very difficult in CKD patients; thus, finding effective measures to reduce IS-induced injury is of significant value.

Previous studies have demonstrated IS-induced tubulointerstitial injury in patients with CKD via the induction of oxidative stress and activation of the NF-κB pathway, producing various cytokines and inflammatory mediators and promoting kidney damage [9–11].

Astragaloside IV (3-O-β-D-xylopyranosyl-6-O-β-D-glucopyranosyl cycloastragenol) is a natural saponin purified from the roots of *Astragalus membranaceus* with antioxidant and anti-inflammatory effects [13, 14]. The anti-inflammatory effects of astragaloside IV have been demonstrated through the inhibition of NF-κB-mediated inflammatory gene expression [24]. Moreover, astragaloside IV exhibits antioxidant effects through the inhibition of reactive oxygen species (ROS) production, reduction of lipid peroxidation, and stimulation of antioxidant enzymes [25]. As mentioned previously, antioxidant treatment strategies are being developed to treat IS-induced tubulointerstitial injury. Oxidative stress is the most critical mechanism of kidney injury induced by indoxyl sulfate, and inflammatory injury is produced after the activation of inflammasome and NF-κB pathway [26]. We speculate that Astragaloside IV pre-intervents the most important oxidative stress step and has weaker pharmacological effects in inflammatory injury induced by indoxyl sulfate in this study.

These studies suggest that astragaloside IV may have a protective effect against IS-induced tubulointerstitial injury and its mechanisms of action may be associated with inhibiting oxidative stress Additional file 1.

## Conclusions

Our study showed that astragaloside IV significantly prevented IS-induced tubulointerstitial injury. Astragaloside IV cannot suppress IS-induced elevation of NF-κB and IL-1β. Treatment with Astragaloside IV remarkably decreased the levels of MDA and 8-OHdG, and simultaneously increased the level of SOD. These results suggest that astragaloside IV could decrease IS-induced oxidative stress, which might constitute an important protective mechanism against tubulointerstitial injury induced by IS.

## Additional file

Additional file 1: Raw Data. (XLSX 16 kb)

## Abbreviations

As-IV: Astragaloside IV
BCA: Bicinchoninic acid
BUN: Blood urea nitrogen
CKD: Chronic kidney disease
DAPI: 4′,6-diamidino-2-phenylindole
ECL: Enhanced chemiluminescent
FITC: Fluoresceine isothiocyanate
HE: Hematoxylin eosin
HPLC: High Performance Liquid Chromatography
IL-1β: Interleukin-1β
IL-6: Interleukin-6
IS: Indoxyl sulfate
NF-κB: Nuclear factor-κB
RIPA: Radio-Immunoprecipitation Assay
SDS-PAGE: dodecyl sulfate, sodium salt-Polyacrylamide gel electrophoresis
